# Landscape Genetic Connectivity and Evidence for Recombination in the North American Population of the White-Nose Syndrome Pathogen, *Pseudogymnoascus destructans*

**DOI:** 10.3390/jof7030182

**Published:** 2021-03-03

**Authors:** Adrian Forsythe, Karen J. Vanderwolf, Jianping Xu

**Affiliations:** 1 Department of Biology, McMaster University, Hamilton, ON L8S 4L8, Canada; adrian.e.forsythe@gmail.com; 2 Department of Environmental and Life Sciences, Trent University, Peterborough, ON K9L 0G2, Canada; kjvanderw@gmail.com; 3 New Brunswick Museum, Saint John, NB E2L 4Z6, Canada

**Keywords:** microsatellite markers, single nucleotide polymorphisms, recombination, landscape genetics, climate, urbanization

## Abstract

White-Nose Syndrome is an ongoing fungal epizootic caused by epidermal infections of the fungus, Pseudogymnoascus destructans (*P. destructans*), affecting hibernating bat species in North America. Emerging early in 2006 in New York State, infections of *P. destructans* have spread to 38 US States and seven Canadian Provinces. Since then, clonal isolates of *P. destructans* have accumulated genotypic and phenotypic variations in North America. Using microsatellite and single nucleotide polymorphism markers, we investigated the population structure and genetic relationships among *P. destructans* isolates from diverse regions in North America to understand its pattern of spread, and to test hypotheses about factors that contribute to transmission. We found limited support for genetic isolation of *P. destructans* populations by geographic distance, and instead identified evidence for gene flow among geographic regions. Interestingly, allelic association tests revealed evidence for recombination in the North American *P. destructans* population. Our landscape genetic analyses revealed that the population structure of *P. destructans* in North America was significantly influenced by anthropogenic impacts on the landscape. Our results have important implications for understanding the mechanism(s) of *P. destructans* spread.

## 1. Introduction

White-Nose Syndrome (WNS) is caused by the fungal pathogen *Pseudogymnoascus destructans* (*P. destructans*) (formerly *Geomyces destructans* [1]. Within hibernating North America (N. America)n bats, infections are characterized by the presence of white mycelial growth on the muzzle or wing tissues of bats, leading to the formation of ulcers and the erosion of epithelial tissues [2]. The N. American *P. destructans* population likely originated from a single European *P. destructans* migrant strain [3,4]. The European *P. destructans* population is known to infect local bat species but has no known associated mortality [5,6]. Since the first recorded case in N. American bats in 2006, *P. destructans* has caused the deaths of millions of bats involving multiple species [7]. Coupled with the increasing bat deaths is the expanding geographic range of WNS in N. America, which is now found in 38 US States and seven Canadian Provinces, including the west coast of N. America [8,9]. Despite the drastic declines in bat populations, relatively little is known about how *P. destructans* and WNS have spread among geographic regions. Because there was very little genetic variation in the initial population of *Pd* when an accidental transmission from Eurasia to North America occurred sometime around 2005/2006, tracking the spread and analyzing the population structure of the N. American *P. destructans* population has been difficult [10,11].

Early investigations into genetic structure of *P. destructans* populations showed results consistent with clonal spread of a single genotype in N. America [12,13,14]. If the pathogen were spread in a step-wise fashion from the center of outbreak origin in New York State, we should expect a relationship between the amount of accumulated mutation during the spread of *P. destructans* and the geographic distance from the site of initial infection. However, recent studies have failed to identify large-scale geographic or temporal structure in the N. American *P. destructans* population [10]. For example, the *P. destructans* isolate from the western-most Washington State was more closely related to the isolate from the epicenter in New York State than some other isolates from within New York State are to the earliest known isolate [11]. However, such a result could be due to chance events in sampling where the genetic relationships between specific isolates may not be representative of the whole populations, and consequently causing misinterpretations of how *P. destructans* has been transmitted between bats and their hibernacula. Alternatively, the spread of *P. destructans* may not follow a strictly step-wise model of outward expansion from the epicenter, but may instead be a more complex processes, influenced by multiple modes of transmission, and both forward and backward migrations between infected regions. In addition, secondary introductions of *P. destructans* genotypes between hibernacula could also influence genetic variation and complicate population structure, making the patterns of genetic variations inconsistent with the step-wise model of clonal expansion. Indeed, the introduction of additional foreign genotypes into N. America could further complicate the population structure and genetic variation. This is especially true if additional introduced strain(s) from Eurasia have a complementary mating type (MAT1-2) to that within the N. American *P. destructans* population (MAT1-1), which could significantly contribute to generating genotypic and phenotypic diversity [15,16].

Much about when and how *P. destructans* spread in N. America remains unknown. Broadly speaking, *P. destructans* could be spread by three methods: primarily via host bats, and secondarily through alternative hosts such as human activities or through passive mechanisms such as wind currents via the dispersal of airborne spores. The majority of WNS spread occurs via the dispersal of *P. destructans* propagules, which are transferred through contact events between hosts. Additional opportunities for transmission may incorporate the movement of bat ectoparasites [17,18], or the spread of propagules by predators of bat species [19], but the extent of their contribution to the spread of WNS may be limited to relatively small geographical distances. In comparison, the distances covered by N. American bats can sometimes extend upwards of 500 km within the migration season [20]. Indeed, the early estimated rate for the expansion of WNS was about 300 km each year, within the range of bats’ movement range within each season [21]. However, the high bat mortality rates (upwards of 90%) associated with *P. destructans* infection in some species result in few surviving individuals within most hibernacula impacted by WNS. Over particularly long distances, the activity of secondary hosts may also contribute to the spread of *P. destructans*. Indeed, human-mediated transmission of *P. destructans* was the most likely cause for *P. destructans* spread from Europe to N. America [22], and it could have continued to contribute to the dispersal of *P. destructans* between caves in N. America [23]. Lastly, the spores of some fungi are known to easily disperse over thousands of kilometers by wind and air currents [24,25]. While short-distance dispersal events of *P. destructans* through the air are unlikely [26], aerosols collected from both inside and outside of hibernacula have been found to contain *P. destructans* spores [27]. Ultimately, the extreme sensitivity of *P. destructans* to temperature and radiation [28,29,30] are likely to further dampen any chance of wind-aided dispersal over extensive distances [31].

The host species impacted by WNS are distributed across a diverse landscape with highly variable climatic conditions. Understanding how landscape features influence the spread of *P. destructans* is an important component of assessing the risk to the spread of further infections. In this study, we employed microsatellite markers and genomic variants to assess the genetic relationships among *P. destructans* isolates and populations, and to identify the potential patterns of dispersal that may exist among the locations sampled across the WNS distribution range in North America. Specifically, we compare the potential influences of landscape features with the null model that geographic distance was the main contributor to genetic distance between isolates. Here, the landscape features that we analyzed include geographical, environmental, or anthropogenic factors. We consider patterns of wind conductance as an additional abiotic factor that may influence connectivity, not as a viable mechanism for the transmission of *P. destructans* in N. America. The relationships of these environmental variables with genotype distributions of *P. destructans* strains across the landscape can also help infer the presence/absence of barriers to *P. destructans* spread. Furthermore, any one of these mechanisms are not mutually exclusive, and may contribute to dispersal depending on local conditions of the hibernacula and/or the surrounding climate. Identifying the dominant influences on the connectivity between bat hibernacula could help formulate targeted approaches to WNS control and prevention within different regions.

## 2. Materials and Methods

### 2.1. Strain Information

The strains analyzed in this study came from two sources: (i) those from published literature that contained genome sequences and/or microsatellite genotype information and (ii) our own strains described in an earlier study [14]. Briefly, our own samples were collected from hibernacula between 2008–2013; the majority were isolated from live bats, cave-associated arthropods, or hibernaculum walls. A few were collected from deceased bats confirmed to have died of WNS. All samples were revived from glycerol freezer stocks held at −80 °C and cultured on nutrient agar media at 14 °C and purified through sub-culturing at the hyphal tip of mycelial growth. These purified cultures matured for one month on Sabouraud Dextrose Agar (SDA) before genomic DNA was extracted using a standard cetyl-trimethylammonium bromide (CTAB) and phenol-chloroform protocol typical for filamentous fungal cultures [32]. Complete strain information is presented in Table S1.

### 2.2. Multilocus Genotyping and Bioinformatic Analyses

For microsatellite genotyping of our own strains, we chose the nine most polymorphic microsatellite loci from the panel developed by [10]. We genotyped 108 isolates using a multiplex PCR method, consisting of three reactions with each reaction containing three sets of primers. In each reaction, each of the three primer sets contained either a HEX, TET, or FAM fluorophore to allow scoring for all three fragments in each reaction. The multiplexed PCR recipe included: 6 μL of 2× GoTaq Green MasterMix (Promega: Madison, Wisconsin), 3 μL nuclease-free water, 0.5 μL of 10 μmol forward primer (total of 1.5 μL for a reaction amplifying three loci), 1 μL of 10 μmol reverse primer (total of 3 μL for a reaction amplifying three loci), and 1 μL of template DNA. PCR products were diluted 10× prior to fragment analysis. All samples were run by Mobix Lab at McMaster University using a GeneAmp PCR SYSTEM 9700 machine (ThermoFisher: Waltham, Massachusetts). The resulting fragment sizes were binned using the Microsatellite Plugin (V1.4, Biomatters: Auckland, New Zealand) for Geneious (4 November 2018), based on the known fragment sizes of isolates in [10] as references.

To further understand the potential geographic patterns of genetic variation in *P. destructans* populations, we analyzed single nucleotide polymorphisms from 41 published genome sequences from the Short Read Archive on NCBI, plus the genomes of three newly sequenced strains [29]. The three new genomes were obtained from strains originating from hibernacula in the Canadian Maritimes and were sequenced on an Illumina MiSeq platform with paired-end libraries. All genomic reads were aligned to the NHWC 20631-21 reference genome [33] using the Burrows-Wheeler Alignment (BWA) bwa mem algorithm (V0.7.15; [34]. We sorted BAM files, removed PCR duplicates, and added read group identifiers using PicardTools (V1.131; [35]. Single Nucleotide Polymorphism (SNP) variants were called from our sample cohort using HaplotypeCaller in gVCF mode (GATK: V4.1.2) at a minimum calling threshold of 20. Variants within repeat-rich regions were discarded after aligning back to the reference genome using NUCmer (V3.23; [36]. Missing calls in our multi-sample VCF were resolved using alignment information in each BAM using FixVcfMissingGenotypes in jvarkit [37]. We excluded variants within our re-sequenced sample of the reference genome and variants with low quality (QUAL > 1000) and depth (DP > 10). The final set of variants included 131 SNPs among 45 N. American *P. destructans* strains (see results below). We added annotations and prediction of effects of SNPs using SnpEff [38].

### 2.3. Population Genetic Analyses

We calculated several population genetic statistics using our microsatellite and SNP datasets. All population genetic statistics were generated using the R package poppr [39]. Our microsatellite dataset consists of 139 isolates, including 108 *P. destructans* isolates we genotyped in this study, plus 31 N. American and 5 European isolates (those with geographical coordinates available) initially genotyped by [10]. For the SNP dataset, we excluded SNP loci if greater than 5% of isolates had no ’REF/ALT’ call. If we identified multiple isolates from the same hibernaculum having the same multilocus genotype, only one of the clonal isolates was used in subsequent population and landscape analyses.

The patterns of allelic relationships among loci were measured using the index of association tests standardized by the number of loci used ($\bar{r}D$). The null hypothesis for index of association measures is that there is random association among alleles at different loci. Statistical significance was derived by comparing the observed index of association to a null distribution (assuming random recombination) over 999 permutations. After a randomization test on a shuffled dataset, simulated *p*-values below 0.05 indicates that the null hypothesis of random recombination should be rejected. To further reveal which pairs of loci might show evidence of recombination, we also measured linkage disequilibrium (LD) between all pairs of loci.

The proportion of pairwise loci that were phylogenetically incompatible, also known as the four-gamete test (FGT) [40], was determined using Multilocus (V1.3; [41]). In short, the FGT considers all possible combinations of alleles between two loci. Loci are assumed to be phylogenetically compatible if there is no evidence of homoplasy or recombination. We considered the results of a FGT against a null distribution of 999 permutations of a shuffled dataset. We used SNP loci to determine genomic regions where potential breakpoints from recombination exist using FGTpartitioner [42]. Lastly, we conducted ϕ tests for recombination in both datasets using SplitsTree4 (V4.13.1) [43]. Population genetic structure was estimated using the STRUCTURE (V2.3.4) and fastSTRUCTURE (V2.7) [44] for microsatellite and SNP datasets, respectively, obtained from N. American isolates and European isolates.

### 2.4. Landscape Genetics

To analyze how landscape features might be associated with genetic relationships among isolates, we first generated genetic distance metrics from our SNP and microsatellite datasets using the first two principal components based on individual-level allele usage [45]. We followed the best practices for resistance surface optimization using the all_comb function in ResistanceGA [46]. Resistance surfaces were created using the commuteDistance algorithm in gdistance [47]. Optimized resistance surfaces were used in a series of Maximum Likihood Population Effects mixed-effects models (MLPE), using the function MLPE.lmm [46]. We considered multiple hypotheses to test the patterns of genetic distance between our sampled sites: (i) Isolation by Distance (IBD) that proposes that gene flow is a function of the Euclidean distance among populations; (ii) Isolation by Environment (IBE) that states that a higher level of gene flow should occur among locations with similar climate; (iii) Isolation by Resistance (IBR) that proposes that gene flow is a function of the resistance distance; and (iv) a null model that assumes the absence of any geographic structure. To evaluate the relative support of the competing models, we assessed each model’s fit using the Akaike information criterion (AIC), and conducted bootstrapping replications by subsampling response and distance matrices, then fitting them back to the MLPE model. The percentage of bootstrap replications in which each IBD, IBE, or IBR model was the best-fitting determines the level of support for each model.

To test the different models, we collected rasters of climate, elevation, anthropogenic impact, and wind conductance across regions overlapping the sampling locations for the isolates that were investigated in this study. The consensus of climatic differences were determined by the combining of 19 bio-climatic variables (worldclim.org, variables “bio1-19”, accessed 18 March 2020) into a single surface using raster principal component analysis (PCA), availible in the R package RStoolbox [48] in order to reduce the amount of multicollinearity between variables. Elevation data were obtained from R package elevatr [49]. We collected raster layers of anthropogenic impact from the Commission for Environmental Cooperation which estimates the impact of human activity across the landscape with an index of human influence; a measure which encapsulates land-use, light pollution, and human density, among others, to gauge the degree of disturbance across the landscape. We generated a raster surface of wind conductance using monthly averages of wind speed and direction with rWind [50]. All variables were aggregated to a resolution of ∼ 20${}^{\prime }$ (∼26 km) prior to optimization.

Lastly, we estimated effective migration rates based on genetic distances using the Estimated Effective Migration Surfaces (EEMS) software package [51]. This method visualizes the influence of IBD across geographic space and creates estimates of effective migration rates by interpolation. Complementary to our MLPE approach using resistance surfaces, EEMS creates effective migration surfaces across space without incorporating environmental variables. We conducted three independent simulations of 200 demes with 1 million burn-in Markov chain Monte Carlo steps over 2 million iterations. Estimated migration surfaces were created from averaging the results of all simulation replicates using the rEEMSplots package [51].

## 3. Results

### 3.1. Genetic Relationships among North American P. destructans

**Figure 1:**
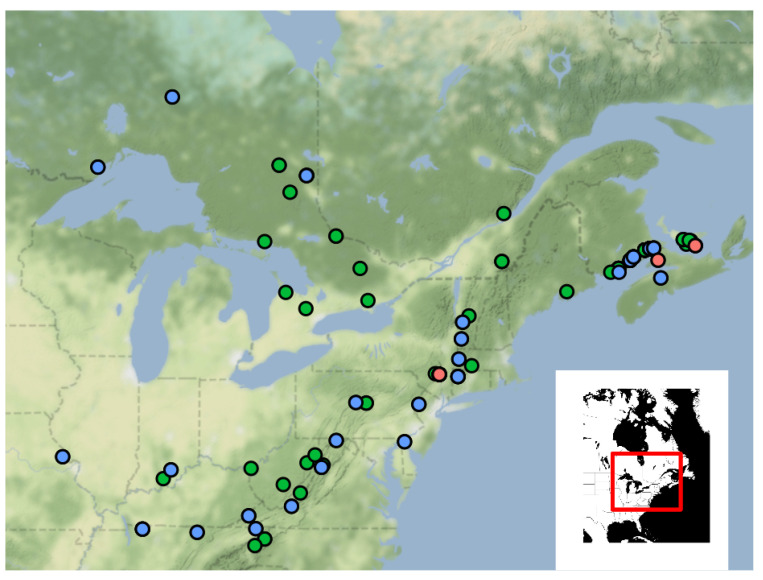
Map of sampling locations from across North America, with each point representing one of more isolate(s) genotyped using whole genome SNPs (blue), multiple microsatellite loci (green), or a combination of both (red). Many of the isolates used here are the result of genotyping in previous studies (See Table S1).

We obtained multilocus microsatellite genotypes from 108 N. American *P. destructans* isolates in our collection using primers targeting nine microsatellite loci (Table S2). These isolates belonged to 42 multilocus genotypes (MLGs). We present these results in combination with the MLG data from [10] to provide a greater context for the genetic relationships among *P. destructans* isolates in N. America (after clonal correction, N=134, MLGs =59; Figure 1). Within this combined dataset, and after correcting for clonal genotypes among different locations, we found an overall high allelic diversity (Simpson’s Diversity; λ=0.98), and well over half the MLGs were exclusive to a single isolate. However, 35 isolates distributed among caves in New Brunswick, Prince Edward Island, New York, Ontario, Québec, and Vermont belonged to a single microsatellite genotype (Table 1). Aside from this dominant genotype, 12 other genotypes were also shared by two or more isolates, often from multiple US States/Canadian Provinces.

Complementary to the nine microsatellite marker data, we also analyzed the genome sequences of 41 N. American *P. destructans* strains. A total of 54 SNPs were identified and these SNPs resolved the 41 strains into 40 SNP genotypes. Two isolates from Glebe and Markhamville Mine in New Brunswick shared an identical SNP genotype. Similar to what we observed within microsatellite genotypes, SNP diversity was high (λ = 0.966, Table 2) compared to previous observations in the N. American population (λ = 0.69) [10]. All SNPs reported here were unique to N. America and have not been found within European *P. destructans* genomes.

### 3.2. Linkage Disequilibrium and Tests for Recombination

**Figure 2:**
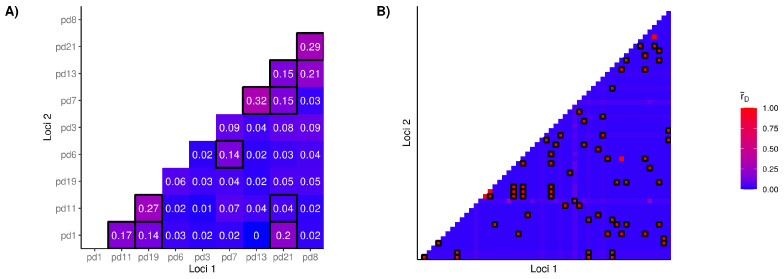
Pairwise comparison of the results from standardized index of association tests ($\bar{r}D$), conducted on Microsatellite (**A**) and SNP (**B**) datasets. Squares of the heatmap outlined in black highlight combinations of loci where $\bar{r}D$ *p*-value < 0.05, consistent with null-expectations under clonality. Samples missing greater than 5% of genotype calls were excluded.

Standardized index of association ($\bar{r}D$) values are often used in tests of LD and are compared to a null distribution of $\bar{r}D$ values, representing linkage equilibrium (LD) as with random recombination in the genome. If the observed is significantly different from the null distribution, the null hypothesis of LD would be rejected and the observed $\bar{r}D$ would be consistent with LD. After censoring clonal isolates, analyses of microsatellite ($\bar{r}D$ = 0.02, *p* = 0.22) and SNP genotypes ($\bar{r}D$ = 0.006, *p* = 0.23) failed to reject the null hypothesis. These tests of index of association were also performed in a hierarchical manner, to identify patterns of linkage between pairs of loci (Table 2). With further partitioning, $\bar{r}D$ showed patterns of association not significantly different from LD in most loci combinations of microsatellite (72%) and SNPs (94%). Only ten of the 36 microsatellite pairs were inconsistent with LD (Figure 2A); whereas 83 of the 1 431 SNP pairs (∼6% of all combinations) were not in LD (Figure 2B). These results demonstrate that most loci are in LD within the N. American *P. destructans* population.

**Figure 3:**
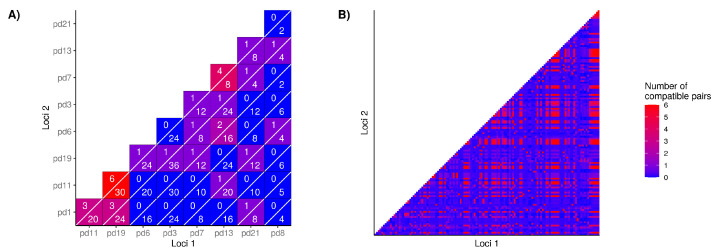
Pairwise comparison of bi-allelic loci where the presence of more than three allele combinations results in phylogenetic incompatibility. Within our microsatellite dataset (**A**) the numerator shows the number of pairs that are compatible, while the denominator lists the total number of allelic combinations between the loci. (**B**) SNP loci with greater than 5% of samples missing calls were dropped.

**Figure 4:**
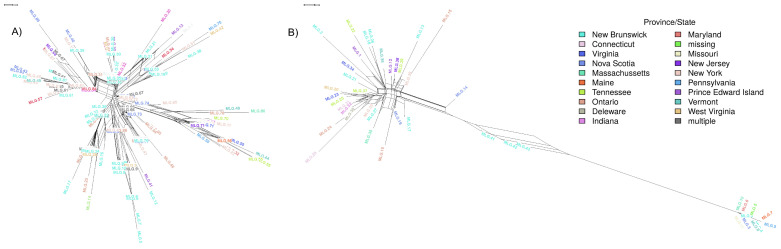
Neighbor-network constructed using SplitsTree using (**A**) 9 microsatellite loci and (**B**) 53 SNP loci. Parallel edges in this network indicate incongruence with a perfect monophyletic tree.

The above index of association and LD tests used random recombination as the null hypothesis to generate the expected genotype counts. We also investigated whether loci pairs are incompatible with strict clonal reproduction in the N. American *P. destructans* population using the FGT. Overall, 50% of microsatellite loci were phylogenetically incompatible (Figure 3A, *p* < 0.01). However, phylogenetic incompatibility was not evenly distributed, as combinations of alleles between some loci (i.e., Pd1/Pd11/Pd19 and Pd7/Pd13) had higher proportions of phyllogenetically compatible alleles (50–12.5%, respectively) compared to others (*p* < 0.01; Figure 3A). Considering all combinations of SNP loci, 25% were inconsistent with phylogenetic compatibility (*p* > 0.05; Figure 3B). Consistent with results from linkage equilibrium and phylogenetic compatibility analyses, the neighbour-network analysis of the N. American *P. destructans* isolates also showed prevalent closed loops (Figure 4) and evidence of recombination in the population. As expected, greater evidence for recombination in European isolates were found. For example, the FGT suggested the presence of four recombination breakpoint events in the N. American *P. destructans* population while 402 breakpoints were estimated in the European population (Figure S1). Taken together, our results indicate unambiguous evidence for recombination in the North American *P. destructans* population.

A neighbour-network with N. American *P. destructans* isolates showed the prevalence of closed loops which indicates incongruence with a perfect monophyletic tree (Figure 4), consistent with recombination and the presence of recombinant lineages in the population. However, ϕ tests for recombination conducted on these collections are not consistent with frequent recombination (SNPs ϕ = 0.138, *p* = 0.9). Whereas, with the inclusion of European strains in these tests, ϕ is consistent with frequent recombination (SNPs ϕ = 0.122, *p* < 0.01). FGT loci suggest the presence of four breakpoint events within the N. American *P. destructans* population, compared to the 402 FGT breakpoints estimated from EU loci in the *P. destructans* genome (Figure S1). Taken together, our results indicate unambiguous evidence for recombination within the N. American *P. destructans* population.

### 3.3. Population Structure

**Figure 5:**
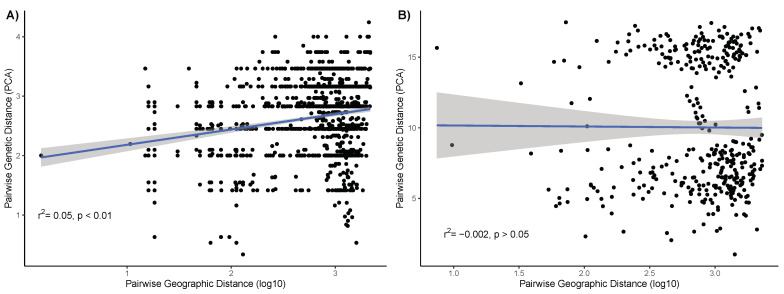
Correlation between genetic distance of (**A**) microsatellites and (**B**) SNPs and logarithm of geographical distance of all pairwise combinations of isolates.

To visualize the population structure of *P. destructans* in N. America, we created unrooted phylogenies using N. American strains genotyped by microsatellites and SNPs, respectively. As revealed by microsatellite genotype information, common genotypes (e.g., MLG 83) and genotype clusters often include isolates from multiple locations (Figure 4). While we detected a statistically significant positive correlation between geographic distance and genetic distance based on microsatellite genotypes, that correlation was very weak ($r^{2}$ = 0.05, *p* > 0.01; Figure 5A). Furthermore, there was no correlation between geographic distance and genetic distance based on SNPs ($r^{2}$ = −0.002, *p* = 0.9; Figure 5B). These results are consistent with frequent gene flows between geographic populations.

Using STRUCTURE, we identified the optimal number of clusters (k=2) for the *P. destructans* genomes analysed in this study from N. America and Europe. Interestingly, the two genotyping methods used here present different patterns in ancestral population structure. Based on microsatellite genotyping, the majority of USA isolates and all European isolates were assigned to one cluster separated from the other cluster that contained mostly Canadian and some US isolates (Figure S2A). In comparison, based on SNP markers, all isolates from Canada and US strains formed a cluster that also contained two isolates from Europe (Germany and Switzerland) while the other cluster contained only European isolates (Figure S2B).

### 3.4. Landscape Genetics

We examined the potential influence of multiple landscape factors on the patterns of *P. destructans* genetic distance in the microsatellite and SNP genotype datasets. We measured the performance of several models to test hypotheses of IBD, IBE, and IBR. The summary results of these tests are presented in Table 3. Interestingly, the model selection results differed between microsatellite and SNP datasets (Table 3). Models incorporating estimates of anthropogenic influence on the landscape (AICc = 1221.26, bootstrap = 88.9%, Table 3) was the best fitting model compared to all others considered for the microsatellite dataset, explaining patterns of genetic distance between individuals better than all other models. Overall, the microsatellite dataset demonstrated that genetic distance increases with greater resistance across human impacted landscapes (slope = 0.16, *t*-value = 10.6). These results suggest that the anthropogenic factors, such as differences in land use and population density, may have prominent impacts on the connectivity between bat hibernacula, and in-turn the genetic similarity between *P. destructans* isolates.

Based on the SNP dataset, the MLPE model with the highest bootstrap support was IBD (51.3%), followed by IBR—Human influence (22.7%), and IBE (21%; Table 3). These results suggested IBD as an important driver of the relationships among strains in N. American *P. destructans*. However, the relationship between genetic and geographic distance is relatively weak (slope = −0.002, *t*-value = −0.01). Furthermore, models suggesting isolation via human influence on the landscape consistently had the lowest average AIC and highest average marginal $r^{2}$ scores compared to all other models tested (Table 3). Together, these analyses also indicate a positive relationship between genetic distance of *P. destructans* strains and resistance to human influence across the landscape (slope = 0.34, *t*-value = 1.26).

**Figure 6:**
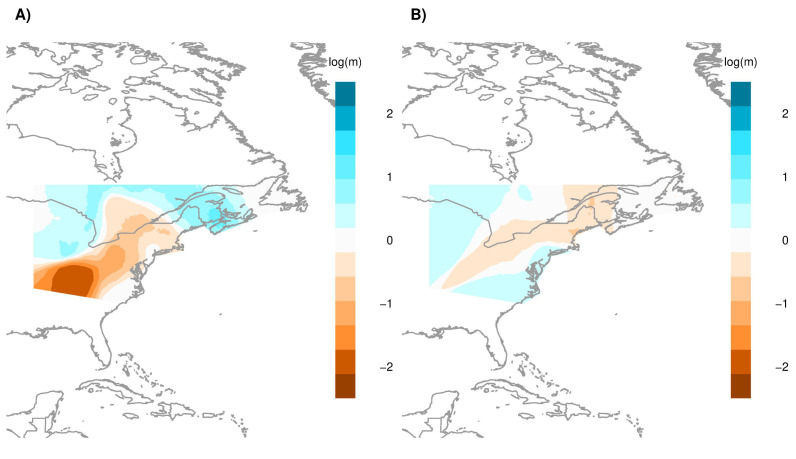
Estimated effective migration surfaces (EEMS) plot for *P. destructans* based on (**A**) SNP and (**B**) MSAT markers. Considering a maximum of 200 demes across the landscape, EEMS generates posterior mean migration rates (log10), indicating areas which have a higher (blue) or lower (orange) rate of migration (shown in blue) than expected under isolation by distance (IBD).

We estimated effective migration surfaces across the landscape for an area roughly $2\times 10^{6}$ ${\mathrm{km}}^{2}$ in size, equivalent to ∼1/5 of the total area of the United States. Figure 6 shows areas of high/low estimated migration rates between locations using both the SNP and microsatellite datasets. Consistent between these datasets is a large section of the distribution with low rates of migration, extending in a south-west/north-east direction (Figure 6A,B). As the estimated migration rates in this region are lower than what would be anticipated under IBD, this suggests reduced gene flow between these areas of the WNS distribution. Flanked by this region are parts of the distribution that have comparably higher estimated rates of migration, greater than expected under the null hypothesis of IBD (Figure 6A,B). Taken together, estimated migration surfaces for both the SNP and microsatellite datasets suggest a disconnect between the south west and north east corners of the distribution.

## 4. Discussion

This study investigated the patterns of genetic variation in the N. American population of *P. destructans*, including the potential landscape factors influencing strain relationships and population structure. Using microsatellite markers and whole-genome sequence data, we found substantial allelic diversity, including novel alleles not reported in prior studies with these markers [10,52]. In addition, we found that the N. American *P. destructans* population is inconsistent with the expectations of strict clonality. We built upon similar recent studies by including a collection of isolates from regions not previously investigated [10,11]. Our analyses revealed limited signals of population genetic structure based on a standard population genetic approach. Our analyses identified connectivity of *P. destructans* isolates strongly influenced by climate and human impacts on landscapes in N. America. Below we discuss the factors influencing the genetic diversity of *P. destructans* across the landscape.

### 4.1. Multilocus Genetic Variation

In clonal microbial pathogens, the rapid generation of novel MLGs via mutation can help overcome selective pressures and niche heterogeneity challenges [53,54,55]. For a recently introduced pathogen that primarily reproduces asexually, we expect the N. American *P. destructans* population to harbor low genotype diversity [56,57]. In comparison, an ancient population capable of both asexual and sexual reproduction would have high genotype diversity, with evidence of loci in LD and phylogenetically incompatible loci [58,59]. Our analyses based on both microsatellite and SNP genotype data are inconsistent with strict asexual reproduction of the N. American *P. destructans* population (Figure 2 and Figure 3). The occurrence of hyphal fusion between members of the same mating type is not uncommon within clonal populations, as parasexual recombination can be a major force of evolution among asexual fungi, causing increased genetic diversity and the emergence of new pathotypes [60]. Many other species of Ascomycota maintain cellular machinery necessary for the parasexual life cycle [61]. Although mitotic recombination has been described within other *Pseudogymnoascus* genera [62], more evidence is needed to distinguish the mechanisms responsible for the allelic patterns observed in this study.

Evidence of recombination has been reported in several seemingly asexual fungal populations where only a single mating type was present (e.g., [63]). A recent study by [64] also suggested evidence of recombination based on sequences from multiple loci in a collection of global *P. destructans* strains. However, they compared their data to only the null model of random recombination. With relatively limited sample size, it might be difficult to reject the null model. In contrast, our analyses based on a much larger sample size compared the data to both the strictly asexual reproduction model and the random recombination model. Thus, we believe that our results provide much more robust support for recombination in the N. American *P. destructans* population.

As different mating types have been found to co-occur within the same ecological niche [4], mating between complementary mating types and sexual reproduction might be common within the ancestral range of *P. destructans* in Europe and Asia. However, only one mating type (MAT1-1) has been reported in the N. American *P. destructans* population (Table S2) [4]. As a result, recombination in the N. American *P. destructans* population unlikely arose through traditional heterosexual mating. Instead, same-sex mating and/or parasexuality, as have been reported for other fungi [65], could have contributed to recombination in the N. American *P. destructans* population. Alternatively, there could be multiple introduction events of strains of the same mating type from Europe where recombination is likely common [66]. More extensive sampling is needed in order to critically evaluate these two possibilities.

The two types of genotype data provided complementary insights into the structure of *P. destructans* in N. America. However, there were several differences worth noting. For example, in this dataset, SNPs were useful for distinguishing European isolates of *P. destructans* and for identifying those from Europe that were closely related to N. American strains (Figure S2B). In addition, patterns of inferred migration differed slightly between the two genotyping methods (Figure 6). Some differences were likely due to the different sample sizes and distributions of the analyzed isolates by the two genotyping methods. Here, more locations were sampled at a higher depth using the microsatellite markers than the SNPs while very few isolates were genotyped using both methods. Furthermore, due to the different mutational processes for the two types of markers, models explaining population structure based on microsatellite markers may be more useful for revealing fine-scale genetic structure while SNP results are more suited for large-scale and long-term inferences.

Indeed, previous studies have suggested that the rate of mutation in microsatellite loci is significantly higher than nucleotide substitutions. Consequently, homoplasy will likely be less common for SNP markers than for microsatellite markers. Here, homoplasy refers to a microsatellite allele or a SNP shared by different strains due to parallel mutational events but not due to their common ancestor. Homoplasy could contribute to LD and phylogenetic incompatibility. While the true rate of homoplasy for either type of markers is unknown in the N. American *P. destructans* population, if present at a significant rate, we expect that the number of phylogenetically incompatible pairs of loci should be higher for loci with a higher number of alleles per locus. However, there is limited statistical support for this hypothesis (Figure 4 and Table S1), suggesting that homoplasy is unlikely to be the major cause of phylogenetic incompatibility and the observed evidence for recombination. Furthermore, we did not find any evidence for the assayed microsatellite markers or the SNPs being in regions of mutational hotspots. For example, we found that none of the SNPs were distributed close to the 1 kb region flanking microsatellite markers based on our whole-genome analyses.

### 4.2. Factors Contributing to Pseudogymnoascus destructans Population Structure

The spread of *P. destructans* in N. America was initially believed to be due to clonal expansion [13]. As such, we would expect *P. destructans* to accumulate genetic diversity as it spread from the epicenter of the outbreak. Overall, we found statistically significant support for a correlation between genetic distance based on microsatellite markers and geographic distance within N. America. However, the correlation coefficient was very weak ($r^{2}$ = 0.05) and many isolates with identical genotypes were shared across distant sampling sites (Figure 5; Table 2). Indeed, almost half of the isolates genotyped using microsatellite markers belong to a single genotype (MLG 83) and this genotype was distributed across several Canadian Provinces/US States (Figure 4 and Table 2). Furthermore, there was no correlation between SNP-based genetic distance and geographical distance among isolates (Figure 5). Taken together, this suggests that gene flow is frequent between sites within regions impacted by WNS.

Our interpretation of *P. destructans* population structure suggests limited correlation with genetic distance (Figure 4 and Table 3). Even through the impact of *P. destructans* infections varies depending on species characteristics [67], the genetic structure of *Myotis lucifugus* has been found to be both consistent [68,69] and inconsistent [70] with the spread of infections. Indeed, the effect of geographic distance on *P. destructans* population structure may be limited [68,71] as long-distance dispersal events push the expansion front and back-transmission fills in behind [72,73].

Climatic patterns influence bat population structure via the seasonal regulation of migration behaviours, impacting when and where host bats congregate in social groups [74,75]. An understanding of the migration patterns of N. American Myotis between winter and summer roosts is crucial for understanding the patterns of WNS transmission [68,76,77,78,79,80,81,82]. Regional similarities in climate appear to be important in regulating the timing of seasonal migrations which may correspond to minimizing energy expenditure [83,84] or prey availability [85]. We report no substantial influence of seasonal variations in climate on the genetic connectivity between *P. destructans* strains (Table 3). Yet, estimated migration rates between sampled locations appear to be consistent with landscape resistance to climate (Figure 6B and Figure S3A); this result indicates that additional climatic measures (or other variables that vary with climate) need to be included to better explain population structure.

Variation in landscape topography or climate is an important driver of the phylogeographic patterns found in a wide variety of taxa [86]. For instance, in Eastern United States and Canada, the Appalachians present a major geological feature that overlaps with bat migratory routes [22]. The high plateau and extending mountain range of the Allegheny Front escarpment may act as a barrier to gene flow, as colonies of little brown bats located on the western side of the Appalachian high plateau were infected with WNS 1–2 years later than colonies in central or eastern Pennsylvania [8,68]. While this feature of the landscape definitely has a local impact on bat populations, we find no influence of altitude on the overall structure of *P. destructans* populations (Table 3). Similarly, while wind dispersal may be a common mechanism of passive dispersal for some fungal species [25,57], we find the genetic connectivity among *P. destructans* isolates is not consistent with prevailing currents (Table 3).

Variation in climate across the landscape could potentially impact the survival of *P. destructans* propagules [29,30] in environmental reservoirs during the summer months. Recent detection of *P. destructans* on free-flying bats during the summer months [23,87] suggests that cells of this fungus can persist on bats for some time. In comparison, positive detection of *P. destructans* appears to be much more common on the skin of European bats [88,89]. As such, either the persistence of *P. destructans* over the summer months or the seasonal shedding and recolonization of the skin microbiome from the environment could enable WNS transmission in N. America. As bat activity decreases at temperatures ≤ 10 °C [90], dormant *P. destructans* cells could then propagate when conditions are favorable [67,91,92].

Human activities could potentially influence the transmission of WNS, by directly facilitating the transmission of *P. destructans* between caves and mines [3,22]. Although commonly isolated from the sediments of caves impacted by WNS [93], *P. destructans* does not appear to be commonly picked up by cave visitors [23,94] and the frequency of human visits to rural caves is unknown. Instead, indirect influences on transmission may be impacted by the consequences of urbanization/land use [95,96].

Our results suggest that areas with high anthropogenic impacts have lower connectivity between locations with regards to *P. destructans* genotypes. Compared to forested areas, both species richness and relative abundance of bats are lower in urban areas [97,98]. At higher latitudes of the WNS distribution, the degree of human impact on the landscape drops off quickly (Figure S3C). Regional migration of bats occurs in all directions, not just latitudinally [99]. For instance, different migration strategies may impact the distance covered by certain species during seasonal migrations. Overwintering at northern latitudes is energetically expensive, and hibernating in social groups is one strategy that can reduce the amount of energy needed to survive the winter [100]. Instead, longer seasonal migrations may take place, in order to reach warmer hibernacula in the south. This has been recorded in *Perimyotis subflavus*, as individuals summering at northern latitudes have been found to migrate farther south [101]. Yet, some *P. subflavus* still overwinter in the hibernacula found at the northern edge of their distribution alongside other bat species [102]. These differing migration strategies may serve as an explanation for why these regions may remain well connected Figure 6 and the long distance dispersal of *P. destructans*.

Although some insectivorous bats do remain in large urban environments [103,104], some Vespertilionidae may have a higher preference for natural areas than urban areas in N. America [105]. With a higher degree of urbanization and fragmentation of suitable habitat, population sizes are decreased [106,107]. As such, high levels of urban development seem to restrict the frequency and length of migration events, reducing the connectivity between populations separated by anthropogenic impact on the landscape [105]. If bat migrations encounter resistance from anthropogenic change to the landscape, then by extension, they can impact the genetic structure of *P. destructans*.

The breadth of sampling for WNS may be limited, as the understanding of where particular environmental sources and sinks exist could be further developed. Much of the least populated areas are less likely to be surveyed as those near city centers. As our analyses suggest higher rates of migration that span areas with lesser human influence, where resistance to migration (and connectivity of *P. destructans* strains) is relaxed (Figure 6), more thorough sampling of these areas could reveal the impact of human activity on the spread of *P. destructans*.

## 5. Conclusions

The increasing prevalence of fungal epidemics emphasizes the importance of understanding how fungal pathogens spread and invade new ecological niches [108]. Rapid and effective genotyping methods are essential for monitoring wildlife diseases, especially WNS, as the high mobility of host species and airborne nature of pathogens themselves present significant challenges in tracking pathogen transmission. In recent years, high-throughput DNA sequencing technologies have improved the accuracy of genetic variation estimates, providing abundant SNPs often with greater discriminating power compared to a few microsatellite markers in most studies [109,110,111]. In this study, we have found that these genetic markers can provide complementary information for inferences about *P. destructans* population structure. Both types of genotype data showed deviations from the null expectations of strict clonality in N. America. In addition, we find that the N. American *P. destructans* population could not strictly be explained a stepping-stone model, involving IBD scenarios of dispersal. Instead, N. American population structure of *P. destructans* has been influenced by anthropogenic impacts across the landscape and IBD and IBE may play secondary roles.

More complex models incorporating seasonal changes across the landscape, including more robust estimates of *P. destructans* prevalence in environments, and measurement of fungal loads on free-flying bats are needed to better understand the population structure of *P. destructans* in N. America.   

## Figures and Tables

**Table 1:** Shared microsatellite genotypes present in multiple caves across North America. The isolates reported here for each genotype have been clone-corrected to exclude clonal individuals from the same sampling site.

MLG Code	Number of Isolates	Province/State, Country	Site
MLG.9	3	New Brunswick, Canada	Glebe Mine
			Markhamville Mine
			White Cave
MLG.11	3	New Brunswick, Canada	Glebe Mine
			Howes Cave
MLG.13	2	New Brunswick, Canada	Glebe Mine
			White Cave
MLG.15	2	West Virginia, USA	Pendleton
			Tucker
MLG.16	2	Nova Scotia, Canada	Falmouth
		New York, USA	Williams Hotel Mine
MLG.25	4	Not Available	Not Available
		North Carolina, USA	Yancey
		Ontario, Canada	Not Available
MLG.33	2	Tennessee, USA	Not Available
		Tennessee, USA	Not Available
MLG.35	2	New Brunswick, Canada	Dorchester Mine
		Prince Edward Island, Canada	Uigg
MLG.40	8	Connecticut, USA	Not Available
		Delaware, USA	Not Available
		Massachusetts, USA	Not Available
		Missouri, USA	Not Available
		Tennessee, USA	Not Available
		Virginia, USA	Not Available
		Not Available	Not Available
MLG.47	7	New Brunswick, Canada	Markhamville Mine
			Not Available
		Not Available	Not Available
MLG.69	2	New Brunswick, Canada	Berryton Cave
			Harbells Cave
MLG.72	7	New Brunswick, Canada	Berryton Cave
			White Cave
		Prince Edward Island, USA	Rocky Point
			Vernon Bridge
MLG.73	3	New Brunswick, Canada	Berryton Cave
			Harbells Cave
			White Cave
MLG.75	6	New Brunswick, Canada	Berryton Cave
			Dorchester Mine
			Markhamville Mine
			White Cave
		Ontario, Canada	Not Available
MLG.80	2	Indiana, USA	Not Available
		West Virginia, USA	Not Available
MLG.82	4	New Brunswick, Canada	Berryton Cave
			Dorchester Mine
			Markhamville Mine
		Prince Edward Island, USA	Murray River
MLG.83	35	New Brunswick, Canada	Berryton Cave
			Dorchester Mine
			Glebe Mine
			Harbells Cave
			Markhamville Mine
			White Cave
		North Carolina, USA	Avery
		New York, USA	Williams Hotel Mine
		Ontario, Canada	Not Available
			Not Available
			Not Available
			Not Available
			Not Available
		Prince Edward Island, Canada	Rocky Point
		Québec, Canada	Not Available
		Virginia, USA	Greely Mine
MLG.84	3	Ontario, Canada	Not Available
		Not Available	Not Available
MLG.87	6	New Brunswick, Canada	Berryton Cave
			Glebe Mine
			Harbells Cave
			Markhamville Mine
			White Cave

**Table 2:** Microsatellite genotypes and population genetic metrics from 139 isolates presented in this study. All metrics were clone-corrected at the Province/State level. Tests of standardized index of association ($\bar{r}D$) are paired with simulated *p*-values generated from a randomization tests, conducted over 1000 permutations of the test statistic.

Region	Country	State/Province	N	MLG	λ ^†^	Hexp ^‡^	$\bar{r}D$	$\bar{r}D$*p*-Value
North America	Canada	New Brunswick	23	23	0.957	0.38	0.065	0.65
		Nova Scotia	1	1	0	-	-	-
		Ontario	11	11	0.91	0.32	−0.013	0.87
		Prince Edward Island	6	6	0.83	0.24	−0.069	0.88
		Québec	2	2	0.5	0.22	-	-
		Total	97 (not clone-corrected)	36	0.97	0.38	0.02	0.89
	USA	Connecticut	1	1	0	-	-	-
		Delaware	1	1	0	-	-	-
		Indiana	2	2	0.5	0.22	-	-
		Massachusetts	2	2	0.5	0.33	-	-
		MD	1	1	0	-	-	-
		ME	1	1	0	-	-	-
		Missouri	1	1	0	-	-	-
		North Carolina	3	3	0.67	0.33	0.83	0.78
		NJ	1	1	0	-	-	-
		New York	5	5	0.8	0.41	0.12	0.12
		OH	1	1	0	-	-	-
		PA	2	2	0.5	0.67	-	-
		Tennessee	3	3	0.67	0.22	0.83	0.77
		Virginia	3	3	0.67	0.37	0.4	0.16
		VT	2	2	0.5	0.44	-	-
		West Virginia	5	5	0.8	0.4	0.12	0.1
		**Total**	37 (not clone-corrected)	26	0.96	0.46	0.04	0.03
	**Total**		134 (not clone-corrected)	59	0.98	0.46	0.03	0.16
Europe	**Total**		5	5	0.8	0.51	0.08	0.27

^†^ = Simpson’s Diversity and ^‡^ = Expected Heterozygosity.

**Table 3:** Assessment of environmental variables contributing to *P. destructans* genotype distributions across landscape. Using a maximum likelihood population effects mixed-model, we measured the effects of various environmental variables/resistance surfaces to predict changes in pairwise genetic differences (PCA scores) generated from microsatellite or SNP alleles. AICc: adjusted Akaike information criterion; weight; marginal $R^{2}$ values of the fitted MLPE model. LL: log likelihood; Percent Top: the percentage of times each model was the best-fit model in over 1000 bootstrap replications.

	Variable(s)	Mean AICc	Mean Weight	Mean Marginal $R^{2}$	Mean LL	Percent Top	k
MSAT	Human Influence	1217.11	0.56	0.08	−604.01	90.5	4
	Climate × Human Influence	1221.43	0.07	0.08	−605.07	0	7
	Wind × Human Influence	1221.53	0.07	0.08	−605.12	0	7
	Climate	1222.17	0.06	0.06	−606.55	0.1	4
	Elevation × Human Influence	1221.64	0.06	0.07	−605.17	0	7
	Wind	1223.34	0.12	0.06	−607.13	9.4	4
	Climate × Elevation	1224.69	0.02	0.06	−606.7	0	7
	Climate × Wind	1225.9	0.02	0.06	−607.3	0	7
	Climate × Elevation × Human Influence	1227.26	0	0.07	−606.08	0	10
	Distance	1227.83	0.01	0.05	−609.76	0	2
	Elevation × Wind	1227.97	0	0.05	−608.34	0	7
	Elevation	1228.45	0.01	0.05	−609.68	0	4
	Climate × Elevation × Wind	1228.86	0	0.06	−606.88	0	10
	Climate × Wind × Human Influence	1228.93	0	0.05	−606.92	0	10
	Elevation × Wind × Human Influence	1229.25	0	0.06	−607.08	0	10
	Climate × Elevation × Wind × Human Influence	1234.15	0	0.07	−606.57	0	13
SNP	Distance	1211.72	0.25	0	−601.52	46.3	2
	Climate	1212.3	0.21	0.02	−600.9	23.7	4
	Human Influence	1212.09	0.24	0.01	−600.8	25	4
	Elevation	1213.18	0.13	0.01	−601.34	4.9	4
	Wind	1213.42	0.11	0	−601.46	0.1	4
	Climate × Human Influence	1218.24	0.01	0.02	−600.81	0	7
	Climate × Wind	1218.57	0.01	0.01	−600.98	0	7
	Wind × Human Influence	1218.4	0.01	0.01	−600.89	0	7
	Climate × Elevation	1218.58	0.01	0.01	−600.98	0	7
	Elevation × Human Influence	1218.43	0.01	0.01	−600.91	0	7
	Elevation × Wind	1219.38	0.01	0	−601.38	0	7
	Climate × Elevation × Human Influence	1231.92	0	0.01	−600.96	0	10
	Climate × Elevation × Wind	1232.13	0	0.01	−601.07	0	10
	Elevation × Wind × Human Influence	1231.96	0	0.01	−600.98	0	10
	Climate × Wind × Human Influence	1232.18	0	0.01	−601.09	0	10
	Climate × Elevation × Wind × Human Influence	1262.07	0	0.01	−601.04	0	13

## Data Availability

Data available in a publicly accessible repository The data presented in this study are openly available on the NCBI SRA (see Table S1).

## Supplementary Materials

The following are available online at https://www.mdpi.com/2309-608X/7/3/182/s1, Table S1: Sample information of the *P. destructans* genomes included in this study. Raw data collected from the NCBI Short Read Archive. Table S2: Summary of basic information on the microsatellite loci used for genotyping, and allelic diversity (Simpson’s index, 1−D). Microsatellite loci originally described by Drees et al. [52] based on the *P. destructans* reference genome (ATCC 20631-21). Figure S1: The estimated genomic locations of recombination breakpoints based on FGT criteria Using SNPs from (A) North American and (B) European samples. Contigs from *P. destructans* reference genome. Gene coding regions in green. Contiguous regions alternate between red and blue at estimated FGT break points. Black points are SNP locations. Figure S2: Visualization of STRUCTURE results in a “distruct” plot, showing the combined membership probability for the optimal number of populations, k=2. The individual membership of each isolates genotyped with A) microsatellite loci (134 North American isolates and 7 European isolates) and B) SNPs (44 North American isolates and 8 European isolates). Figure S3: Optimized landscape surfaces for (A) climate, (B) elevation, (C) human influence index, and (D) wind conductance.

## Abbreviations

AIC	Akaike information criterion
CTAB	cetyl-trimethylammonium bromide
FGT	four-gamete test
IBD	Isolation by Distance
IBE	Isolation by Environment
IBR	Isolation by Resistance
LD	linkage disequilibrium
LE	linkage equilibrium
MLG	multilocus genotype
MLPE	Maximum Likihood Population Effects mixed-effects models
N. America	North America
*P. destructans*	*Pseudogymnoascus destructans*
SDA	Sabouraud Dextrose Agar
SNP	Single Nucleotide Polymorphism
WNS	White-Nose Syndrome

## References

[1] Gargas A., Trest M.T., Christensen M., Volk T.J., Blehert D.S. (2009). *Geomyces destructans* sp. nov. associated with bat White-Nose syndrome. Mycotaxon.

[2] Meteyer C.U., Buckles E.L., Blehert D.S., Hicks A.C., Green D.E., Shearn-bochsler V., Thomas N.J., Gargas A., Behr M.J. (2009). Histopathologic criteria to confirm White-Nose syndrome in bats. J. Vet. Diagn. Investig..

[3] Leopardi S., Blake D., Puechmaille S.J. (2015). White-Nose Syndrome fungus introduced from Europe to North America. Curr. Biol..

[4] Palmer J.M., Kubatova A., Novakova A., Minnis A.M., Kolarik M., Lindner D.L. (2014). Molecular Characterization of a Heterothallic Mating System in *Pseudogymnoascus destructans*, the Fungus Causing White-Nose Syndrome of Bats. G3-Genes. Genom. Genet..

[5] Hoyt J.R., Langwig K.E., Sun K., Parise K.L., Li A., Wang Y., Huang X., Worledge L., Miller H., White J.P. (2020). Environmental reservoir dynamics predict global infection patterns and population impacts for the fungal disease white-nose syndrome. Proc. Natl. Acad. Sci. USA.

[6] Fritze M., Puechmaille S.J. (2018). Identifying unusual mortality events in bats: A baseline for bat hibernation monitoring and white-nose syndrome research. Mammal Rev..

[7] Froschauer A., Coleman J. (2012). North American Bat Death Toll Exceeds 5.5 Million from White-Nose Syndrome.

[8] Heffernan A. (2017). White-Nose Syndrome Map. https://www.whitenosesyndrome.org/static-spread-map.

[9] Lorch J.M., Palmer J.M., Lindner D.L., Ballmann A.E., George K.G., Griffin K., Knowles S., Huckabee J.R., Haman K.H., Anderson C.D. (2016). First Detection of Bat White-Nose Syndrome in Western North America. mSphere.

[10] Drees K.P., Parise K.L., Rivas S.M., Felton L.L., Bastien S., Puechmaille J., Keim P., Foster J.T. (2017). Characterization of Microsatellites in *Pseudogymnoascus destructans* for White-nose Syndrome Genetic Analysis. J. Wildl. Dis..

[11] Trivedi J., Lachapelle J., Vanderwolf K.J., Misra V., Willis C.K.R., Ratcliffe J.M., Ness R.W., Anderson J.B., Kohn L.M. (2017). Fungus Causing White-Nose Syndrome in Bats Accumulates Genetic Variability in North America with No Sign of Recombination. mSphere.

[12] Ren P., Haman K.H., Last L.A., Rajkumar S.S., Kevin Keel M., Chaturvedi V. (2012). Clonal spread of *Geomyces destructans* among bats, Midwestern and Southern United States. Emerg. Infect. Dis..

[13] Rajkumar S.S., Li X., Rudd R.J., Okoniewski J.C., Xu J., Chaturvedi S., Chaturvedi V. (2011). Clonal genotype of *Geomyces destructans* among bats with White Nose Syndrome, New York, USA. Emerg. Infect. Dis..

[14] Khankhet J., Vanderwolf K.J., McAlpine D.F., McBurney S., Overy D.P., Slavic D., Xu J. (2014). Clonal expansion of the *Pseudogymnoascus destructans* genotype in North America is accompanied by significant variation in phenotypic expression. PLoS ONE.

[15] Halkett F., Simon J.C., Balloux F. (2005). Tackling the population genetics of clonal and partially clonal organisms. Trends Ecol. Evol..

[16] Arnaud-Haond S., Migliaccio M., Diaz-Almela E., Teixeira S., Van De Vliet M.S., Alberto F., Procaccini G., Duarte C.M., Serrao E.A. (2007). Vicariance patterns in the Mediterranean Sea: East–west cleavage and low dispersal in the endemic seagrass *Posidonia oceanica*. J. Biogeogr..

[17] Lučan R.K., Bandouchova H., Bartonička T., Pikula J., Zahradníková A., Zukal J., Martínková N. (2016). Ectoparasites may serve as vectors for the white-nose syndrome fungus. Parasites Vectors.

[18] Vanderwolf K.J., Malloch D., McAlpine D.F. (2016). Ectomycota Associated with Arthropods from Bat Hibernacula in Eastern Canada, with Particular Reference to *Pseudogymnoascus destructans*. Insects.

[19] McAlpine D.F., Vanderwolf K.J., Forbes G.J., Malloch D. (2011). Consumption of bats (Myotis spp.) by raccoons (Procyon lotor) during an outbreak of white-nose syndrome in New Brunswick, Canada: Implications for estimates of bat mortality. Can. Field-Nat..

[20] Norquay K.J.O., Martinez-Nuñez F., Dubois J.E., Monson K.M., Willis C.K.R. (2013). Long-distance movements of little brown bats (*Myotis lucifugus*). J. Mammal..

[21] Foley J., Clifford D., Castle K., Cryan P., Ostfeld R.S. (2011). Investigating and managing the rapid emergence of White-Nose syndrome, a novel, fatal, infectious disease of hibernating bats. Conserv. Biol..

[22] Reynolds H.T., Barton H.A. (2013). White-Nose Syndrome: Human Activity in the Emergence of an Extirpating Mycosis. Microbiol. Spectr..

[23] Ballmann A.E., Torkelson M.R., Bohuski E.A., Russell R.E., Blehert D.S. (2017). Dispersal hazards of *Pseudogymnoascus destructans* by bats and human activity at hibernacula in summer. J. Wildl. Dis..

[24] Aylor D.E. (1990). The role of intermittent wind in the dispersal of fungal pathogens. Annu. Rev. Phytopathol..

[25] Rieux A., Soubeyrand S., Bonnot F., Klein E.K., Ngando J.E., Mehl A., Ravigne V., Carlier J., de Lapeyre de Bellaire L. (2014). Long-distance wind-dispersal of spores in a fungal plant pathogen: Estimation of anisotropic dispersal kernels from an extensive field experiment. PLoS ONE.

[26] Lorch J.M., Meteyer C.U., Behr M.J., Boyles J.G., Cryan P.M., Hicks A.C., Ballmann A.E., Coleman J.T.H., Redell D.N., Reeder D.M. (2011). Experimental infection of bats with *Geomyces destructans* causes White-Nose syndrome. Nature.

[27] Kokurewicz T., Ogórek R., Pusz W., Matkowski K. (2016). Bats Increase the Number of Cultivable Airborne Fungi in the “Nietoperek” Bat Reserve in Western Poland. Microb. Ecol..

[28] Palmer J.M., Drees K.P., Foster J.T., Lindner D.L. (2018). Extreme sensitivity to ultraviolet light in the fungal pathogen causing White-Nose syndrome of bats. Nat. Commun..

[29] Forsythe A., Giglio V., Asa J., Xu J. (2018). Phenotypic divergence along geographic gradients reveals potential for rapid adaptation of the White-nose Syndrome pathogen, *Pseudogymnoascus destructans*, in North America. Appl. Environ. Microbiol..

[30] Campbell L.J., Walsh D.P., Blehert D.S., Lorch J.M. (2020). Long-Term Survival of *Pseudogymnoascus destructans* at Elevated Temperatures. J. Wildl. Dis..

[31] Hayes M.A. (2012). The *Geomyces* Fungi: Ecology and Distribution. Bioscience.

[32] Katz M.E., Cheetham B.F., Liu D. (2009). Isolation of Nucleic Acids from Filamentous Fungi. Handbook of Nucleic Acid Purification.

[33] Drees K.P., Palmer J.M., Sebra R., Lorch J.M., Chen C., Wu C.C., Bok W., Keller N.P., Blehert D.S., Cuomo C.A. (2016). Use of Multiple Sequencing Technologies To Produce a High-Quality Genome of the Fungus *Pseudogymnoascus destructans*, the Causative Agent of Bat White-Nose Syndrome. Genome Announc..

[34] Li H., Durbin R. (2010). Fast and accurate long-read alignment with Burrows-Wheeler transform. Bioinformatics.

[35] Picard Toolkit (2019). Broad Institute, GitHub Repository. http://broadinstitute.github.io/picard.

[36] Delcher A.L., Salzberg S.L., Phillippy A.M. (2003). Using MUMmer to identify similar regions in large sequence sets. Curr. Protoc. Bioinform..

[37] Lindenbaum P. (2015). JVarkit: Java-based utilities for Bioinformatics. FigShare.

[38] Cingolani P., Platts A., Wang L.L., Coon M., Nguyen T., Wang L., Land S.J., Lu X., Ruden D.M. (2012). A program for annotating and predicting the effects of single nucleotide polymorphisms, SnpEff: SNPs in the genome of Drosophila melanogaster strain w1118; iso-2; iso-3. Fly.

[39] Kamvar Z.N., Tabima J.F., Grünwald N.J. (2014). Poppr: An R package for genetic analysis of populations with clonal, partially clonal, and/or sexual reproduction. PeerJ.

[40] Hudson R.R., Kaplan N.L. (1985). Statistical properties of the number of recombination events in the history of a sample of DNA sequences. Genetics.

[41] Agapow P.M., Burt A. (2001). Indices of multilocus linkage disequilibrium. Mol. Ecol. Notes.

[42] Chafin T.K. (2019). FGTpartitioner: Parsimonious delimitation of ancestry breakpoints in large genome-wide SNP datasets. bioRxiv.

[43] Huson D.H., Bryant D. (2006). Application of phylogenetic networks in evolutionary studies. Mol. Biol. Evol..

[44] Raj A., Stephens M., Pritchard J.K. (2014). fastSTRUCTURE: Variational inference of population structure in large SNP data sets. Genetics.

[45] Kierepka E.M., Latch E.K. (2015). Performance of partial statistics in individual-based landscape genetics. Mol. Ecol. Resour..

[46] Peterman W.E. (2018). ResistanceGA: An R package for the optimization of resistance surfaces using genetic algorithms. Methods Ecol. Evol..

[47] Van Etten J. (2017). R Package gdistance: Distances and Routes on Geographical Grids. J. Stat. Softw.

[48] Leutner B., Horning N. (2017). RStoolbox: Tools for Remote Sensing Data Analysis. https://bleutner.github.io/RStoolbox/.

[49] Hollister J., Shah T. (2017). Elevatr: Access Elevation Data from Various APIs. https://github.com/jhollist/elevatr/.

[50] Fernández-López J., Schliep K. (2019). rWind: Download, edit and include wind data in ecological and evolutionary analysis. Ecography.

[51] Petkova D., Novembre J., Stephens M. (2016). Visualizing spatial population structure with estimated effective migration surfaces. Nat. Genet..

[52] Drees K.P., Lorch J.M., Puechmaille S.J., Parise K.L., Wibbelt G., Hoyt J.R., Sun K., Jargalsaikhan A., Dalannast M., Palmer J.M. (2017). Phylogenetics of a Fungal Invasion: Origins and Widespread Dispersal of White-Nose Syndrome. mBio.

[53] Monis P.T., Caccio S.M., Thompson R.C.A. (2009). Variation in *Giardia*: Towards a taxonomic revision of the genus. Trends Parasitol..

[54] Campbell L.T., Carter D.A. (2006). Looking for sex in the fungal pathogens *Cryptococcus neoformans* and *Cryptococcus gattii*. FEMS Yeast Res..

[55] Narra H.P., Ochman H. (2006). Of What Use Is Sex to Bacteria?. Curr. Biol..

[56] Raboin L.M., Selvi A., Oliveira K.M., Paulet F., Calatayud C., Zapater M.F., Brottier P., Luzaran R., Garsmeur O., Carlier J. (2007). Evidence for the dispersal of a unique lineage from Asia to America and Africa in the sugarcane fungal pathogen *Ustilago scitaminea*. Fungal Genet. Biol..

[57] Hovmøller M.S., Yahyaoui A.H., Milus E.A., Justesen A.F. (2008). Rapid global spread of two aggressive strains of a wheat rust fungus. Mol. Ecol..

[58] Attanayake R.N., Tennekoon V., Johnson D.A., Porter L.D., del Río-Mendoza L., Jiang D., Chen W. (2014). Inferring outcrossing in the homothallic fungus *Sclerotinia sclerotiorum* using linkage disequilibrium decay. Heredity.

[59] Sibley L.D., Ajioka J.W. (2008). Population structure of *Toxoplasma gondii*: Clonal expansion driven by infrequent recombination and selective sweeps. Annu. Rev. Microbiol..

[60] Read N.D., Roca M.G. (2006). Vegetative hyphal fusion in filamentous fungi. Cell-Cell Channels.

[61] Glass N.L., Jacobson D.J., Shiu P.K. (2000). The genetics of hyphal fusion and vegetative incompatibility in filamentous ascomycete fungi. Annu. Rev. Genet..

[62] Leushkin E.V., Logacheva M.D., Penin A.A., Sutormin R.A., Gerasimov E.S., Kochkina G.A., Ivanushkina N.E., Vasilenko O.V., Kondrashov A.S., Ozerskaya S.M. (2015). Comparative genome analysis of Pseudogymnoascus spp. reveals primarily clonal evolution with small genome fragments exchanged between lineages. BMC Genom..

[63] Hiremath S., Lehtoma K. Ectomycorrhizal fungi association with the American chestnut 2006. https://www.srs.fs.usda.gov/pubs/12330.

[64] Sharma L., Sousa M., Faria A.S., Nunes-Pereira M., Cabral J.A., Phillips A.J., Marques G., das Neves Paiva-Cardoso M. (2019). Worldwide recombination in emergent white-nose syndrome pathogen Pseudogymnoascus destructans. BioRxiv.

[65] Ene I.V., Bennett R.J. (2014). The cryptic sexual strategies of human fungal pathogens. Nat. Rev. Microbiol..

[66] Dilmaghani A., Gladieux P., Gout L., Giraud T., Brunner P.C., Stachowiak A., Balesdent M.H., Rouxel T. (2012). Migration patterns and changes in population biology associated with the worldwide spread of the oilseed rape pathogen *Leptosphaeria maculans*. Mol. Ecol..

[67] Langwig K.E., Frick W.F., Bried J.T., Hicks A.C., Kunz T.H., Kilpatrick A.M. (2012). Sociality, density-dependence and microclimates determine the persistence of populations suffering from a novel fungal disease, White-Nose syndrome. Ecol. Lett..

[68] Miller-Butterworth C.M., Vonhof M.J., Rosenstern J., Turner G.G., Russell A.L. (2014). Genetic structure of little brown bats (*Myotis lucifugus*) corresponds with spread of White-Nose syndrome among hibernacula. J. Hered..

[69] Burns L.E., Frasier T.R., Broders H.G. (2014). Genetic connectivity among swarming sites in the wide ranging and recently declining little brown bat (*Myotis lucifugus*). Ecol. Evol..

[70] Davy C.M., Martinez-Nunez F., Willis C.K.R., Good S.V. (2015). Spatial genetic structure among bat hibernacula along the leading edge of a rapidly spreading pathogen. Conserv. Genet..

[71] Vonhof M.J., Russell A.L., Miller-Butterworth C.M. (2015). Range-Wide Genetic Analysis of Little Brown Bat (*Myotis lucifugus*) Populations: Estimating the Risk of Spread of White-Nose Syndrome. PLoS ONE.

[72] Wilder A.P., Frick W.F., Langwig K.E., Kunz T.H. (2011). Risk factors associated with mortality from White-Nose syndrome among hibernating bat colonies. Biol. Lett..

[73] Thogmartin W.E., Andrew King R., Szymanski J.A., Pruitt L. (2012). Space-time models for a panzootic in bats, with a focus on the endangered Indiana bat. J. Wildl. Dis..

[74] Biek R., Real L.A. (2010). The landscape genetics of infectious disease emergence and spread. Mol. Ecol..

[75] Warnecke L., Turner J.M., Bollinger T.K., Lorch J.M., Misra V., Cryan P.M., Wibbelt G., Blehert D.S., Willis C.K.R. (2012). Inoculation of bats with European *Geomyces destructans* supports the novel pathogen hypothesis for the origin of White-Nose syndrome. Proc. Natl. Acad. Sci. USA.

[76] Park K.J., Jones G., Ransome R.D. (2000). Torpor, arousal and activity of hibernating Greater Horseshoe Bats (*Rhinolophus ferrumequinum*). Funct. Ecol..

[77] Smith A.D., McWilliams S.R. (2016). Bat activity during autumn relates to atmospheric conditions: Implications for coastal wind energy development. J. Mammal..

[78] Jonasson K.A. (2017). The Effects of Sex, Energy, and Environmental Conditions on the Movement Ecology of Migratory Bats. Ph.D. Thesis.

[79] Pettit J.L., O’Keefe J.M. (2017). Impacts of White-Nose Syndrome Observed During Long-Term Monitoring of a Midwestern Bat Community. J. Fish Wildl. Manag..

[80] Reynolds H.T., Ingersoll T., Barton H.A. (2015). Modeling the environmental growth of *Pseudogymnoascus destructans* and its impact on the White-Nose syndrome epidemic. J. Wildl. Dis..

[81] Krauel J.J., McGuire L.P., Boyles J.G. (2018). Testing traditional assumptions about regional migration in bats. Mammal Res..

[82] Taylor P.D., Mackenzie S.A., Thurber B.G., Calvert A.M., Mills A.M., McGuire L.P., Guglielmo C.G. (2011). Landscape Movements of Migratory Birds and Bats Reveal an Expanded Scale of Stopover. PLoS ONE.

[83] Adams R.A., Hayes M.A. (2008). Water availability and successful lactation by bats as related to climate change in arid regions of western North America. J. Anim. Ecol..

[84] Popa-Lisseanu A.G., Voigt C.C. (2009). Bats on the Move. J. Mammal..

[85] Meyer A.D., Stevens D.F., Blackwood J.C. (2016). Predicting bat colony survival under controls targeting multiple transmission routes of White-Nose syndrome. J. Theor. Biol..

[86] Shafer A.B.A., Cullingham C.I., Côté S.D., Coltman D.W. (2010). Of glaciers and refugia: A decade of study sheds new light on the phylogeography of northwestern North America. Mol. Ecol..

[87] Carpenter G.M., Willcox E.V., Bernard R.F., Stiver W.H. (2016). Detection of *Pseudogymnoascus destructans* on free-flying male bats captured during summer in the southeastern USA. J. Wildl. Dis..

[88] Bandouchova H., Bartonicka T., Berkova H., Brichta J., Cerny J., Kovacova V., Kolarik M., Köllner B., Kulich P., Martínková N. (2015). *Pseudogymnoascus destructans*: Evidence of virulent skin invasion for bats under natural conditions, Europe. Transbound. Emerg. Dis..

[89] Pikula J., Amelon S.K., Bandouchova H., Bartonička T., Berkova H., Brichta J., Hooper S., Kokurewicz T., Kolarik M., Köllner B. (2017). White-nose syndrome pathology grading in Nearctic and Palearctic bats. PLoS ONE.

[90] US Fish and Wildlife Service (2018). Range-Wide Indiana Bat Summer Survey Guidelines.

[91] Marroquin C.M., Lavine J.O., Windstam S.T. (2017). Effect of Humidity on Development of *Pseudogymnoascus destructans*, the Causal Agent of Bat White-Nose Syndrome. Northeast. Nat..

[92] Lilley T.M., Anttila J., Ruokolainen L. (2018). Landscape structure and ecology influence the spread of a bat fungal disease. Funct. Ecol..

[93] Lorch J.M., Muller L.K., Russell R.E., O’Connor M., Lindner D.L., Blehert D.S. (2013). Distribution and environmental persistence of the causative agent of White-Nose syndrome, *Geomyces destructans*, in bat hibernacula of the eastern United States. Appl. Environ. Microbiol..

[94] Morisak K.M. (2017). Variation of *Pseudogymnoascus destructans* Spore Loads and Risk of Human Vectored Transport. Ph.D. Thesis.

[95] Mathews F., Roche N., Aughney T., Jones N., Day J., Baker J., Langton S. (2015). Barriers and benefits: Implications of artificial night-lighting for the distribution of common bats in Britain and Ireland. Philos. Trans. R. Soc. Lond. B Biol. Sci..

[96] Cryan P.M. (2011). Wind Turbines as Landscape Impediments to the Migratory Connectivity of Bats. Environ. Law.

[97] Avila-Flores R., Fenton M.B. (2005). Use of spatial features by foraging insectivorous bats in a large urban landscape. J. Mammal..

[98] Jung K., Kalko E.K. (2011). Adaptability and vulnerability of high flying Neotropical aerial insectivorous bats to urbanization. Divers. Distrib..

[99] Davis W.H., Hitchcock H.B. (1965). Biology and migration of the bat, *Myotis lucifugus*, in New England. J. Mammal..

[100] Boyles J., Storm J., Brack V. (2008). Thermal benefits of clustering during hibernation: A field test of competing hypotheses on Myotis sodalis. Funct. Ecol..

[101] Fraser E.E., McGuire L.P., Eger J.L., Longstaffe F.J., Fenton M.B. (2012). Evidence of latitudinal migration in tri-colored bats, *Perimyotis subflavus*. PLoS ONE.

[102] Vanderwolf K.J., McAlpine D.F. (2021). Hibernacula microclimate and declines in overwintering bats during an outbreak of white-nose syndrome near the northern range limit of infection in North America. Ecol. Evol..

[103] Dixon M.D. (2012). Relationship between land cover and insectivorous bat activity in an urban landscape. Urban Ecosyst..

[104] Duchamp J.E., Swihart R.K. (2008). Shifts in bat community structure related to evolved traits and features of human-altered landscapes. Landsc. Ecol..

[105] Jung K., Threlfall C.G., Voigt C., Kingston T. (2016). Urbanisation and Its Effects on Bats—A Global Meta-Analysis. Bats in the Anthropocene: Conservation of Bats in a Changing World.

[106] Sendor T., Simon M. (2003). Population dynamics of the pipistrelle bat: Effects of sex, age and winter weather on seasonal survival. J. Anim. Ecol..

[107] Papadatou E., Ibáñez C., Pradel R., Juste J., Gimenez O. (2011). Assessing survival in a multi-population system: A case study on bat populations. Oecologia.

[108] De Hoog G.S., Ahmed S.A., Danesi P., Guillot J., Gräser Y., Seyedmousavi S., de Hoog G., Guillot J., Verweij P. (2018). Distribution of Pathogens and Outbreak Fungi in the Fungal Kingdom. Emerging and Epizootic Fungal Infections in Animals.

[109] Santure A.W., Stapley J., Ball A.D., Birkhead T.R., Burke T., Slate J. (2010). On the use of large marker panels to estimate inbreeding and relatedness: Empirical and simulation studies of a pedigreed zebra finch population typed at 771 SNPs. Mol. Ecol..

[110] Helyar S.J., Hemmer-Hansen J., Bekkevold D., Taylor M.I., Ogden R., Limborg M.T., Cariani A., Maes G.E., Diopere E., Carvalho G.R. (2011). Application of SNPs for population genetics of nonmodel organisms: New opportunities and challenges. Mol. Ecol. Resour..

[111] Fischer M.C., Rellstab C., Leuzinger M., Roumet M., Gugerli F., Shimizu K.K., Holderegger R., Widmer A. (2017). Estimating genomic diversity and population differentiation—An empirical comparison of microsatellite and SNP variation in *Arabidopsis halleri*. BMC Genom..

