# Supplementary material for: Landscape Genetic Connectivity and Evidence for Recombination in the North American Population of the White-Nose Syndrome Pathogen, Pseudogymnoascus destructans
Source: J Fungi (Basel). 2021 Mar 3;7(3):182. doi: 10.3390/jof7030182 (PMC8001231; doi:10.3390/jof7030182)
Supplement: Supplementary file 1 [file jof-07-00182-s001.pdf]

Supplementary Materials: Landscape Genetic Connectivity and Evidence for Recombination in the North American Population of the White-Nose Syndrome Pathogen, *Pseudogymnoascus destructans*

Adrian Forsythe [1,†], Karen J Vanderwolf [2,3] and Jianping Xu [1,*]

Table S1. Sample information of the *P. destructans* genomes included in this study. Raw data collected from the NCBI Short Read Archive.

Region	Country	Province/State	Sampling Location	BioProject	Run	Year	Isolation Source
Europe	France	Aquitaine	Dordogne	PRJNA400587	SRR6011497	2009	*Myotis myotis*
	Germany	Thueringen	Gipskarstlandschaft Questenberg	PRJNA400587	SRR6011467	2009	*Myotis myotis*
	Hungary	Kislod	Not Available	PRJNA400587	SRR6011465	2009	*Myotis myotis*
	Not Available	Not Available	Not Available	PRJNA391811	SRR5755629	2009	*Myotis myotis*
	Switzerland	Aargau	Not Available	PRJNA400587	SRR6011468	2009	*Myotis myotis*
	Ukraine	Not Available	Not Available	PRJNA400587	SRR6011496	2011	*Myotis myotis*
North America	Canada	New Brunswick	Dorchester Mine	PRJNA400587	SRR6011488	2012	substrate (cave wall)
				PRJNA472378	NB26_S1	2012	*Myotis lucifugus*
			Harbells Cave	This Study	NB28_S3	Not Available	Not Available
		Nova Scotia	Dorchester Mine	This Study	NB27_S2	Not Available	Not Available
			Falmouth	This Study	NS1_S5	Not Available	Not Available
		Ontario	Cochrane	PRJNA400587	SRR6011483	2010	*Myotis lucifugus*
			Kirkland Lake	PRJNA400587	SRR6011472	2010	*Myotis lucifugus*
			Rattlesnake Point	This Study	ON14_S7	Not Available	Not Available
		Prince Edward Island	Murray River	PRJNA472378	PE8_S6	2012	*Myotis lucifugus*
	Not Available	Not Available	Not Available	PRJNA391811	SRR5755634	2008	*Myotis lucifugus*
					SRR5755633	2013	*Exechiopsis sp.*
					SRR5755632	2013	*Perimyotis subflavus*
					SRR5755631	2013	*Perimyotis subflavus*
					SRR5755630	2013	*Nelima elegans*
					SRR5755628	2012	*Myotis lucifugus*
					SRR5755627	2012	*Myotis lucifugus*
					SRR5755624	2015	Not Available
					SRR5755623	2015	*Myotis lucifugus*
					SRR5755622	2013	*Myotis lucifugus*
					SRR5755621	2013	*Myotis lucifugus*
					SRR5755620	2013	*Myotis lucifugus*
					SRR5755619	2015	*Myotis lucifugus*
					SRR5755618	2015	*Myotis lucifugus*
					SRR5757331	2012	*Myotis lucifugus*
	USA	Connecticut	Litchfield County	PRJNA400587	SRR6011478	2008	*Myotis lucifugus*
					SRR6011475	2009	*Myotis lucifugus*
		Delaware	New Castle County	PRJNA400587	SRR6011494	2012	*Myotis septentrionalis*
		Indiana	Washington County	PRJNA400587	SRR6011493	2011	*Myotis lucifugus*
			Crawford County	PRJNA400587	SRR6011492	2011	*Myotis lucifugus*
		Maine	Hancock County	PRJNA400587	SRR6011495	2011	*Myotis lucifugus*
		Maryland	Allegany County	PRJNA400587	SRR6011480	2010	*Myotis septentrionalis*
		Massachusetts	Berkshire County	PRJNA400587	SRR6011477	2008	*Myotis septentrionalis*
			Hampden County	PRJNA400587	SRR6011466	2008	*Myotis lucifugus*
		Missouri	Lincoln County	PRJNA400587	SRR6011489	2012	*Perimyotis subflavus*
		New Jersey	Warren County	PRJNA400587	SRR6011470	2009	*Myotis lucifugus*
		New York	Ulster County	PRJNA400587	SRR6011481	2009	*Eptesicus fuscus*
		New York	Williams Hotel	PRJNA276926	SRR1952982	2008	*Myotis lucifugus*
		Pennsylvania	Centre County	PRJNA400587	SRR6011469	2009	*Myotis lucifugus*
		Tennessee	Montgomery County	PRJNA400587	SRR6011491	2011	*Myotis lucifugus*
			Fentress County	PRJNA400587	SRR6011484	2010	*Myotis septentrionalis*
			Sullivan County	PRJNA400587	SRR6011479	2010	*Perimyotis subflavus*
			Montgomery County	PRJNA400587	SRR6011473	2010	*Myotis septentrionalis*
		Vermont	Rutland County	PRJNA400587	SRR6011482	2010	*Myotis lucifugus*
			Bennington County	PRJNA400587	SRR6011474	2008	*Myotis septentrionalis*
		Virginia	Wise County	PRJNA400587	SRR6011490	2011	*Myotis lucifugus*
			Giles	PRJNA400587	SRR6011476	2009	Not Available
		West Virginia	Pendleton County	PRJNA400587	SRR6011471	2009	*Myotis septentrionalis*

Table S2. Summary of basic information on the microsatellite loci used for genotyping, and allelic diversity (Simpson's index, $1 - D$). Microsatellite loci originally described by Drees et al [1] based on the *P. destructans* reference genome (ATCC 20631-21).

Locus	Contig (Accession)	Number of Alleles	1-D
Pd1	KV441387	4	0.34
Pd11	KV441394	4	0.31
P19	KV441397	5	0.22
Pd3	KV441391	3	0.3
Pd6	KV441390	6	0.33
Pd7	KV441406	1	-
Pd8	KV441398	2	2
Pd13	KV441393	1	-
Pd21	KV441412	1	-
	**Mean**	3	8

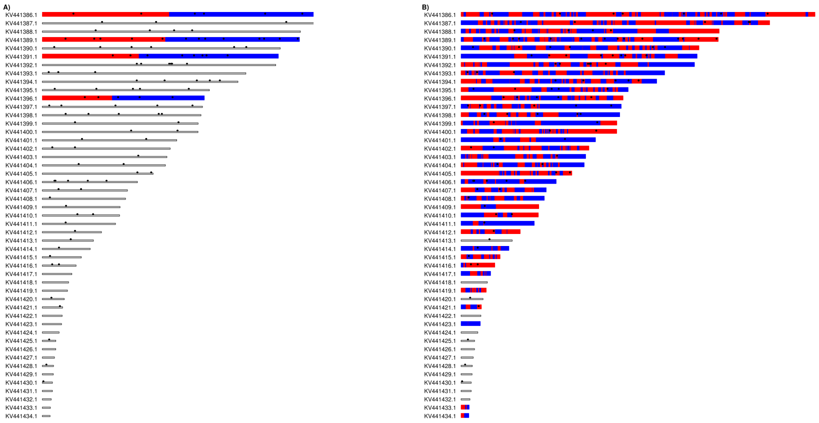

Figure S1. The estimated genomic locations of recombination breakpoints based on four-gamete test criteria Using SNPs from A) North American and B) European samples. Contigs from *Pseudogymnoascus destructans* reference genome. Gene coding regions in green. Contiguous regions alternate between red and blue at estimated FGT break points. Black points are SNP locations.

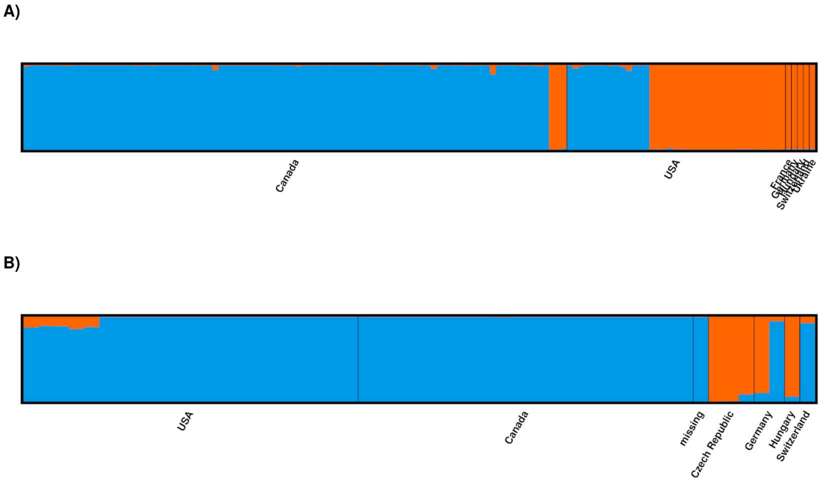

Figure S2. Visualization of `STRUCTURE` results in a "distruct" plot, showing the combined membership probability for the optimal number of populations, $k = 2$. The individual membership of each isolates genotyped with A) microsatellite loci (134 North American isolates and 7 European isolates) and B) SNPs (44 North American isolates and 8 European isolates).

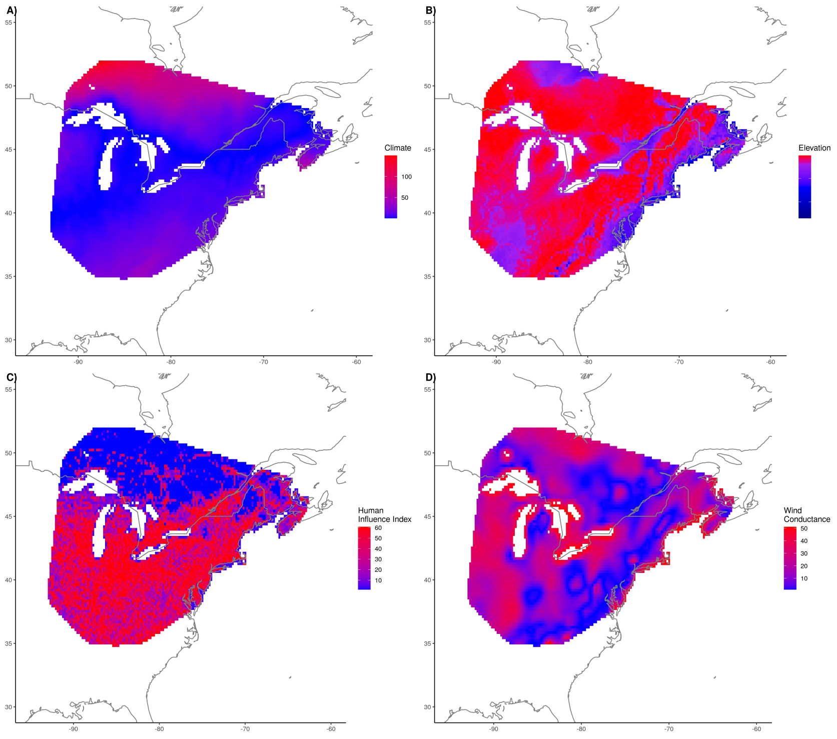

Figure S3. Optimized landscape surfaces for A) climate, B) elevation, C) human influence index, and D) wind conductance.

Supplementary References

1. Drees, K.P.; Lorch, J.M.; Puechmaille, S.J.; Parise, K.L.; Wibbelt, G.; Hoyt, J.R.; Sun, K.; Jargalsaikhan, A.; Dalannast, M.; Palmer, J.M.; Lindner, D.L.; Marm Kilpatrick, A.; Pearson, T.; Keim, P.S.; Blehert, D.S.; Foster, J.T. Phylogenetics of a Fungal Invasion: Origins and Widespread Dispersal of White-Nose Syndrome. *MBio* **2017**, *8*, e01941–17.
